# Impact of selected genetic factors on clopidogrel inactive metabolite level and antiplatelet response in patients after percutaneous coronary intervention

**DOI:** 10.1007/s43440-020-00197-w

**Published:** 2020-12-03

**Authors:** Urszula Adamiak-Giera, Anna Czerkawska, Szymon Olędzki, Mateusz Kurzawski, Krzysztof Safranow, Maria Jastrzębska, Barbara Gawrońska-Szklarz

**Affiliations:** 1 Department of Pharmacokinetics and Therapeutic Drug Monitoring, Pomeranian Medical University, Szczecin, Poland; 2 Department of Cardiology, Pomeranian Medical University, Szczecin, Poland; 3 Department of Experimental and Clinical Pharmacology, Pomeranian Medical University, Szczecin, Poland; 4 Department of Biochemistry and Medical Chemistry, Pomeranian Medical University, Szczecin, Poland; 5 Department of Laboratory Diagnostics and Molecular Medicine, Pomeranian Medical University, Szczecin, Poland

**Keywords:** Clopidogrel inactive metabolite, Concentration, Genetic polymorphisms

## Abstract

**Background and objective:**

Clopidogrel is frequently used as part of optimal dual antiplatelet therapy in high-bleeding risk patients with the acute coronary syndrome. The concentration of the inactive carboxylic acid metabolite of clopidogrel might be useful to evaluate the response to clopidogrel therapy. Therefore, we sought to correlate the inhibition of platelet aggregation with the plasma level of the inactive metabolite of clopidogrel in patients after percutaneous coronary interventions (PCI) and their associations with the most frequently studied genetic polymorphisms. For this purpose, the fast and simple HPLC method for determining the concentration of the inactive metabolite was developed.

**Methods:**

The effect of *CYP2C19*, *CYP3A4/5*, *ABCB1* and *PON1* genes on the plasma inactive metabolite concentration of clopidogrel and the platelet aggregation was investigated in 155 patients before and after PCI.

**Results:**

The concentration of the inactive metabolite of clopidogrel was not significantly different in the intermediate metabolizers (IM) of CYP2C19 compared with extensive metabolizers (EM) both before and after PCI, while inhibition of platelet aggregation was found to be significantly better in EM than in IM. The presence of the A allele at position 2677 in the *ABCB1* gene was associated with a significantly lower concentration of inactive metabolite of clopidogrel before PCI.

**Conclusion:**

The CYP2C19*2 allele was associated with decreased platelet reactivity during clopidogrel therapy before and after PCI. Simultaneous determination of platelet aggregation and concentration of the inactive clopidogrel metabolite may be useful in clinical practice to find the cause of adverse effects or insufficient treatment effect in patients chronically treated with clopidogrel.

## Introduction

Current guidelines recommend potent platelet inhibition with ticagrelor or prasugrel in patients after an acute coronary syndrome [1–3]. However, optimal dual antiplatelet therapy strategy in high-bleeding risk patients presenting with acute coronary syndrome remains debated. Gragnano et al. [4] showed that clopidogrel, rather than potent P2Y12 inhibitors, is frequently used of as part of dual antiplatelet therapy in real-world high-bleeding risk patients presenting with acute coronary syndromes. Although current guidelines do not recommend the use of clopidogrel over ticagrelol in high bleeding risk patients presenting with acute coronary syndrome, several registries have shown that clopidogrel is frequently preferred by clinicians in this setting [4–7]. As shown in clinical trials clopidogrel effectively lowers the occurrence of myocardial infarction, ischemic stroke, and vascular death in patients [7–9]. Despite the obvious advantages of clopidogrel, many clinical studies have shown that approximately 5–40% of patients treated with standard doses of clopidogrel display inadequate antiplatelet responses [10, 11]. A range of 30–90% of variability noted in the pharmacokinetics and pharmacodynamics, particularly absorption and metabolism, are considered attributable to pharmacogenomic variation [10, 11]. Current evidence demonstrates that adherence to secondary prevention medication in acute coronary syndromes is suboptimal across the globe in terms of both persistence with treatment and compliance with dosing instructions throughout the recommended duration of treatment. Non-adherence to oral antiplatelet medications is associated with worse outcomes or failure of therapy [12].

Clopidogrel is a pro-drug that has to be converted to an active metabolite by hepatic cytochrome P450 (CYP) isoenzymes to inhibit platelet aggregation. It is also subject to efflux via P-glycoprotein (encoded by ABCB1, also known as MDR1). ABCB1 polymorphism may affect drug transport and efficacy. Approximately 85% of the orally administered drug is hydrolyzed by esterases in the blood into an inactive carboxylic acid derivative. The remaining drug (about 15%) is bioactivated in a two-step oxidative process by CYP450 isoenzymes in the liver to an active metabolite that selectively and irreversibly inhibits the ADP P2Y12 receptor on platelet cell [10, 13, 14]. A number of functional polymorphisms of the CYP450 enzymes have been shown to affect the metabolism of clopidogrel. The CYP2C19 enzyme plays an important role in the hepatic bioactivation of clopidogrel. A large number of clinical studies have documented that a loss-of-function variant CYP2C19*2 affects clinical profiles of clopidogrel (efficacy and safety). Moreover, the presence of the CYP2C19*17 variant might lead to an increased response to clopidogrel, but carriers of the variant have an increased risk of developing bleeding as well [10, 15–18]. The activation of clopidogrel is partly governed by the CYP3A system, which consists of 3A4 and 3A5 isoenzymes [10, 16]. According to Kazui et al. [14] CYP3A4 is responsible for the transformation of 39.8% of the intermediate product of clopidogrel. Therefore, altered activity of CYP3A4 resulting from genetic polymorphisms might have an influence on the yield of active metabolite formation and on the antiplatelet effect of clopidogrel. Clopidogrel metabolism can also be influenced by genetic variants in other proteins, such as ABCB1, paraoxonase-1 (PON1), carboxylesterase (CES1), and P2Y12 receptors [10, 13, 17–20]. These genetic variants can interfere with the regulation of gene transcription, protein translation, or substrate affinity. As shown in the literature, Q192R (A576G) genetic variant of paraoxonase-1 (*PON1*), an esterase synthesized in the liver and present in the serum, was reported to be strongly involved in clopidogrel bioactivation and clopidogrel responsiveness [19, 20]. These results were in contrast to the Fontana et al. [21] who reported no relationship of the PON1 (Q192R) polymorphism with clinical effects of clopidogrel. Campo et al. [22] demonstrated that PON1 Q192R genotype has a minimal influence on-clopidogrel platelet reactivity variation over time. Other genetic polymorphisms occurring in the gene sequences are also considered as potential factors of the variable response of clopidogrel. Furthermore, polymorphisms in the *ABCB1* may influence cell efflux of clopidogrel, resulting in varied drug bioavailability and response [10, 23, 24]. As shown, *ABCB1 3435 TT* genotype might be associated with lower exposition to the parent drug [24].

The active metabolite of clopidogrel is a very labile molecule that degrades rapidly in blood after sample collection [25], therefore determination of the concentration of the inactive carboxylic acid metabolite of clopidogrel might be useful to evaluate the response to clopidogrel therapy. Serebruany et al. [26] showed that the plasma level of inactive carboxylic acid metabolite but not the level of the active metabolite of clopidogrel is a useful marker for monitoring compliance to clopidogrel in registries and clinical trials.

We aimed to investigate the contribution of the most studied *CYP2C19, CYP3A4/5, PON1,* and *ABCB1* polymorphisms to the plasma level of an inactive carboxylic acid metabolite of clopidogrel and antiplatelet response. For this purpose, the fast and simple HPLC method for determining the concentration of the metabolite was developed. We wanted to assess whether the level of inactive carboxylic acid metabolite determined in one sample at steady-state can be used as a marker to evaluate the antiplatelet effect of clopidogrel in patients after PCI and patients’ compliance with clopidogrel therapy.

## Experimental

### Patients and sample collection

The study included 155 patients (45 female, 110 male) and was conducted in the Department of Cardiology with Intense Cardiac Care (Pomeranian Medical University in Szczecin, Poland). Presented prospective research was approved by the ethics committee at Pomeranian Medical University in Szczecin and written informed consent was obtained from all subjects. Patients received oral clopidogrel as a 75 mg maintenance dose and 75 mg acetylsalicylic acid (ASA) for minimum 7 days prior to the percutaneous coronary intervention procedure (PCI). The persons qualified for the study applied for the performance of the coronarography within the prescribed period. 24 h after this study, a sample of whole peripheral blood was collected at 200 μL to determine selected polymorphisms of the genes *CYP2C19, CYP3A4, CYP3A5, ABCB1,* and *PON1*.

On the established day of PCI procedure, the patients who were enrolled in the study took clopidogrel 75 mg and acetylsalicylic acid 75 mg. Blood samples for quantification of plasma concentrations inactive metabolite of clopidogrel were collected at 2 h after oral administration of a tablet containing 75 mg clopidogrel. A 4 mL of blood was drawn into collection systems containing K_2_EDTA as the anticoagulant and then were centrifuged at 1500 × *g* for 10 min. Obtained plasma samples were stored at − 25 °C until analysis.

At the same time, blood was taken to determine platelet aggregation. Then a PCI procedure was performed. Depending on the indications, patients received a metal stent (BMS) or antiproliferative drug-eluting stent (DES). In the case of BMS stent implantation, the patient was reported after 6 months for an outpatient follow-up visit, continuing double antiplatelet therapy (clopidogrel 75 mg/day + ASA 75 mg/day). After implantation of the DES the patient was reporting after 12 months. On the day of the follow-up visit, a blood sample was being taken to determine the concentration of the metabolite of clopidogrel and at the same time, the reactivity of platelets was examined.

### Determination of inactive metabolite concentration of clopidogrel

Few high-performance liquid chromatographic (HPLC) methods for determination of carboxylic acid metabolite of clopidogrel have been reported in the literature [27–29].

A sensitive LC–MS method was also reported for determination of clopidogrel metabolite in plasma, however LC–MS instrument is not yet readily available in all laboratories. The HPLC method with UV detection is the most convenient and common analytical method. The HPLC analysis of inactive metabolite clopidogrel was performed on a chromatograph HP 1100 with UV detector at a wavelength of 225 nm. Chromatographic separation was achieved by a LiChroCart^®^ 125–4 LiChrispher^®^ 100 Rp-18e (5 µm) column (Merck). The isocratic mobile phase pumped at a flow rate 1 mL/min consisted 0.01 M KH_2_PO_4_ and acetonitrile (70:30 v/v) adjusted to pH 3.0 by phosphoric acid. All separations were performed at 22 °C temperature. The carboxylic acid metabolite of clopidogrel was purchased from Pharmaceutical Research Institute (Warsaw, Poland). Piroxicam (PRX, internal standard, IS) was purchased from Sigma-Aldrich.

Stock standard solutions of the carboxylic acid metabolite of clopidogrel and IS were prepared in methanol at 1 mg/mL concentrations. Then, standard solutions of 4.0, 10.0, 20.0, 40.0, 60.0, 80.0 and 160.0 µg/mL of carboxylic acid metabolite of clopidogrel and 500 µg/mL IS were prepared also in methanol. The volume of 50 µl carboxylic acid metabolite of clopidogrel and 50 µl IS was transferred to a vial containing 0.9 mL human blank plasma. The resulting plasma samples contained 0.2, 0.5, 1.0, 2.0, 3.0, 4.0, 8.0 µg/mL carboxylic acid metabolite of clopidogrel. The plasma samples applied on C_18_ bonded SPE cartridges and absorbed analytes were eluted with 1 mL of methanol. The organic layer was transferred to a clean test tube and evaporated to dryness at 40 °C in a water bath under a steam of nitrogen. The residue was reconstituted in 100 µl mobile phase and 25 µl were injected into the chromatographic system. Designed HPLC method with UV detector was developed and validated for the determination of non-active metabolite of clopidogrel in plasma. The retention times for carboxylic acid metabolite of clopidogrel and IS were 1.9 and 8.9 min respectively and did not interfere with peaks from any drugs co-administered with clopidogrel. The method was linear in the ranges of 0.2–4.0 µg/mL in plasma for the carboxylic acid metabolite of clopidogrel with a correlation coefficient (*r*^2^) of 99.93–99.99%. Limit of detection (LOD) of the carboxylic acid metabolite of clopidogrel was determined as an S/N baseline ratio of 3:1 for plasma samples and it was found to be 0.025 µg/mL. The value of the limit of quantitaion (LOQ) in plasma for the carboxylic acid metabolite of clopidogrel was 0.1 µg/mL. The intra- and inter-assay precision values were ≤ 5.98%. The intra- and inter-day accuracy of the method was ≤ 3.75%. Recovery of carboxylic acid metabolite of clopidogrel from plasma samples was in a range of 85.76–96.91%.

Simple sample preparation procedure and a short chromatographic run time make this method suitable for rapid and reliable determination of clopidogrel metabolite in plasma.

### Pharmacodynamic assay

The pharmacodynamic response was estimated by an impedance method (Multiplate analyzer; Dynabyte medical, Germany). Samples for the assay platelet aggregation in 155 patients who received 75 mg of clopidogrel and 75 mg of ASA were collected 2 h after clopidogrel administration into hirudin-coated S Monovette systems (SarstedtAG&Co., Germany). The ADP-induced platelet aggregation test was performed in accordance with the manufacturer’s instruction. Platelet aggregation was quantified as the area under the curve (AUC_agr_) of the units vs. time (AU·min). According to the consensus opinion of Bonello et al. [30], a cut-off point of 500 AU·min for platelet aggregation in response to ADP should be used as the threshold for an increased risk of thrombotic events during clopidogrel therapy. Patients with AUC_agr_ values > 500 AU·min could be characterized as “non-responders” for clopidogrel therapy.

### Determination of genetic polymorphisms

Genomic DNA was extracted from 200 µL of whole blood samples using GeneMATRIX Quick Blood DNA Purification Kit (EURx, Poland). Each sample was genotyped for a presence of the most frequent SNPs within *CYP2C19* (rs4244285, *2 allele, and rs12248560, *17 allele),*CYP3A4* (rs35599367, *22 allele), *CYP3A5* (rs776746, *3 allele), *ABCB1* (rs1045642, 3435C > T, rs2032582, 2677G > A,T, and rs1128503, 1236T > C), and *PON1* (rs662, Q192R) genes. Prevalidated allelic discrimination TaqMan real-time PCR assays (Life Technologies, USA) were used for genotype determination. Fluorescence data was captured using an 7500 FAST Real-Time PCR System (Life Technologies, USA), after 40 cycles of PCR.

### Statistical analysis

The statistical analysis was performed using Statistica version 10.0 software. Wilcoxon signed-rank test was used to study differences between quantitative variables measured before and after PCI while McNemar’s test was used for paired comparison of qualitative variables. The Mann–Whitney’s *U* test was applied to evaluate the influence of genotypes on the concentration of carboxylic acid metabolite of clopidogrel and pharmacodynamic response before and after PCI. Correlations between the concentration of inactive metabolite and platelet aggregation were calculated with the Spearman correlation coefficient (Rs). A general linear model (GLM) was used for multivariate analysis, where dependent variables were normalized by logarithmic transformation. A *p* value of < 0.05 was considered significant.

## Results

### Patient characteristics

Demographic characteristics, clinical information of the patients are shown in Table 1.

**Table 1:** Patients demographics and patients characteristics

Characteristic	Female *n* = 45 (mean ± SD)	Male *n* = 110 (mean ± SD)
Age (years)	63.36 ± 9.09	61.65 ± 8.17
Height (cm)	158.49 ± 5.65	173.15 ± 5.72
Body weight (kg)	69.52 ± 14.00	84.48 ± 12.18
BMI	27.20 ± 5.48	28.12 ± 3.33

	*n*—Total number of patients
Clopidogrel 75 mg	155
Acid acetylsalicylic 75 mg	155
Simvastatin	14
Rosuvastatin	36
Atorvastatin	97
Omeprazol	6
Pantoprazol	90
Angiotensin-converting enzyme inhibitor	128
Angiotensin receptor blocker	13
Calcium channel blocker	13
Beta blocker	147
Diuretic	47
Diabetes mellitus	44
Hyperlipidaemia	105
Smoking	53

### Concentrations of the inactive metabolite of clopidogrel and platelet aggregation in patients before and after PCI

The concentrations of the inactive metabolite of clopidogrel and platelet aggregation are presented in Table 2. The plasma concentration of inactive metabolite of clopidogrel in 155 patients in a sample taken 2 h after double antiplatelet therapy before PCI, varied substantially among patients from 0.04 to 5.73 μg/mL (mean 1.20). After stent implantation, only 100 patients completed the study, respectively 73 patients with metal stent (BMS) after 6 months and 27 patients with antiproliferative drug-eluting stent (DES) after 12 months. After PCI the plasma concentration of inactive metabolite of clopidogrel were 1.15 (0.17–3.24) μg/mL and 1.10 (0.36–1.67) μg/mL after BMS and DES stents, respectively. The results of the Wilcoxon signed-rank test showed that the plasma concentration of inactive metabolite of clopidogrel before and after PCI were not significantly different (*p* = 0.86 and 0.36 for BMS and DES stents, respectively).

**Table 2:** Concentrations of the inactive metabolite of clopidogrel (C) and platelet aggregation (AUC_agr_) in patients before and after PCI

Parameter	Before PCI *n* = 155	After	PCI
		6 months *n* = 73	12 months *n* = 27
C ± SD (μg/mL) (min–max)	1.20 ± 0.89 (0.04–5.73)	1.15 ± 0.66 (0.17–3.24)	1.10 ± 0.35 (0.36–1.67)
AUC_agr_ ± SD (AU·min) (min–max)	305.75 ± 191.42 (42–1275)	309.66 ± 176.23 (51–1108)	305.67 ± 252.72 (55–1137)

The inter-subject variability in platelet reactivity was observed before and after PCI. The results of platelet tests performed 2 h after double antiplatelet therapy were 305.75 (42–1275) AU·min before PCI, and 309.66 (59–1108) and 305.67 (55–1137) AU·min after BMS and DES stents, respectively. Platelet aggregation before PCI was not statistically significantly different from the value after PCI (*p* = 0.96 and 0.87 for BMS and DES stents, respectively). Taking into account a cut-off point of 500 AU·min for platelet aggregation in response to ADP as the threshold for “responders” and “non-responders” to the clopidogrel therapy, the increased numbers of “responders” were observed after stent implantation and double antiplatelet therapy. Among 155 patients, before stent implantation, 23 (14.8%) could be characterized as “non-responders” with AUC_agr_ values > 500 AU·min, while platelet aggregation in 132 (85.2%) patients exhibited AUC values ≤ 500 AU·min. These patients were classified as “responders” to clopidogrel therapy. After stent implantation and antiplatelet maintenance therapy (100 analyzed patients) the proportion of patients with high reactivity of AUC_agr_ values > 500 AU·min was 12% and the number of patients with AUC_agr_ values ≤ 500 AU·min was 88%. However, no statistically significant differences between the results before and after PCI were observed (*p* = 0.80, McNemar’s test).

No statistically significant correlations were found between the inactive metabolite concentration of clopidogrel and aggregation tests before and after PCI (Spearman’s rank correlation coefficients: Rs = − 0.04, *p* = 0.61, and Rs = − 0.05, *p* = 0.61, respectively).

### Effect of genetic polymorphisms on the inactive metabolite concentration of clopidogrel and platelet aggregation in patients before and after PCI

Genotype distribution of all investigated SNPs was in Hardy–Weinberg equilibrium. Table 3 shows the association of *CYP2C19* genotypes with clopidogrel metabolite concentration and platelet aggregation in patients before and after PCI. Patients with *1/*1, *1/*17 and *17/*17 of *CYP2C19* genotypes were classified as extensive metabolizers (EM). Participants with *1/*2 and *2/*17 of CYP2C19 genotypes were classified as intermediate metabolizers (IM). There was no one with a *2/*2 genotype. Among 155 patients 115 (74.2%) were EM and 40 (25.8%) IM. In the 100 patients who completed the study after PCI, there were 70 (70%) EM and 30 (30%) IM. The concentration of the inactive metabolite of clopidogrel was not significantly different in the intermediate metabolizers compared with the group of extensive metabolizers both before and after PCI.

**Table 3:** Clopidogrel inactive metabolite concentration and platelet aggregation in patients with *CYP2C19* genotype before and after PCI

Parameter	Genotype *CYP2C19*		*p* (Mann–Whitney *U* test)
	*1/*1 + *1/*17 + *17/*17 (EM)	*1/*2 + *2/*17 (IM)	
Before PCI	*N* = 115 (74.2%)	*N* = 40 (25.8%)	
C (μg/mL)	1.18 ± 0.78 (0.06–5.74)	1.25 ± 1.19 (0.07–6.66)	NS
AUC_agr_ (AU·min)	289.26 ± 180.65 (42–1275)	353.15 ± 214.89 (110–1275)	0.018
After PCI	*N* = 70 (70%)	*N* = 30 (30%)	
C (μg/mL)	1.17 ± 0.62 (0.05–3.24)	1.05 ± 0.53 (0.05–2.78)	NS
AUC_agr_ (AU·min)	262.60 ± 146.38 (51–1137)	415.87 ± 257.67 (82–1137)	0.0006

*n* number of patients, *NS* not statistically significant *p* > 0.05

AUC_agr_—platelet aggregation; C—clopidogrel inactive metabolite concentration

Mean ± SD and (Min–Max) values are presented

Inhibition of platelet aggregation (AUCagr) was found to be significantly better in EM than in IM, before (*p* < 0.018) as well as after (*p* < 0.0006) PCI. Despite the fact that in the IM group there were 15 patients with genotype *2/*17.

Tables 4 and 5 show the association of *CYP3A4* and *CYP3A5* genotypes with clopidogrel metabolite concentration and platelet aggregation in patients before and after PCI. In the studied group, there were no significant differences of inactive metabolite concentration of clopidogrel and platelet aggregation between patients with *CYP3A4* and *CYP3A5* genotypes.

**Table 4:** Clopidogrel metabolite concentration (μg/mL) and platelet aggregation (AU·min) in patients with genotype *CYP3A4* before and after PCI

Parameter	Genotype *CYP3A4*		*p* (Mann–Whitney *U* test)
	*1/*1	*1/*22	
Before PCI	*n* = 149 (96.1%)	*n* = 6 (3.9%)	
C (μg/mL)	1.15 ± 0.76 (0.04–3.78)	2.44 ± 2.33 (0.77–6.66)	NS
AUC_agr_ (AU·min)	298.93 ± 173.01 (42–946)	475.00 ± 454.63 (61–1275)	NS
After PCI	*n* = 97 (97%)	*n* = 3 (3%)	
C (μg/mL)	1.13 ± 0.60 (3.12–3.24)	1.32 ± 0.48 (0.97–1,86)	NS
AUC_agr_ (AU·min)	304.17 ± 191.71 (51–1137)	451.33 ± 391.05 (64–846)	NS

*n* number of patients, *NS* not statistically significant *p* > 0.05

AUC_agr_—platelet aggregation (AU·min); C—clopidogrel metabolite concentration (μg/mL)

Mean ± SD and (Min–Max) values are presented

**Table 5:** Clopidogrel metabolite concentration (μg/mL) and platelet aggregation (AU·min) in patients with genotype *CYP3A5* before and after PCI

Parameter	Genotype *CYP3A5*		*p* (Mann–Whitney *U* test)
	*1/*1 + *1/*3	*3/*3	
Before PCI	*n* = 15 (9.7%)	*n* = 140 (90.3%)	
C (μg/mL)	1.04 ± 0.87 (0.07–3.53)	1.21 ± 0.90 (0.04–6.66)	NS
AUC_agr_ (AU·min)	340.67 ± 190.65 (131–743)	302.01 ± 191.80 (42–1275)	NS
After PCI	*n* = 12 (12%)	*n* = 88 (88%)	
C (μg/mL)	1.07 ± 0.48 (0.20–1.92)	1.14 ± 0.61 (3.12–3.24)	NS
AUC_agr_ (AU·min)	391.17 ± 307.15 (105–1137)	297.32 ± 178.32 (51–1108)	NS

*n* number of patients, *NS* not statistically significant *p* > 0.05

AUC_agr_—platelet aggregation (AU·min); C—clopidogrel metabolite concentration (μg/mL)

Mean ± SD and (Min–Max) values are presented

Tables 6, 7 and 8 show the association of *ABCB1 3435C* > *T,2677C* > *T* and *1236C* > *T* genotypes with clopidogrel metabolite concentration and platelet aggregation in patients before and after PCI. There were no significant differences in the concentrations of the metabolite of clopidogrel and platelet aggregation in the studied patients with individual genotypes of *ABCB1* 3435C > T and *ABCB1* 1236C > T polymorphisms. The presence of the A allele at position 2677 in the *ABCB1* gene was associated with a significantly lower concentration of the metabolite of clopidogrel (*p* = 0.026) before PCI when carriers and non-carriers of the A allele were compared. This difference was not observed after stent implantation, but it should be noted that the statistical power for this comparison is very small due to the small number of patients with the A allele examined after PCI (*n* = 2).

**Table 6:** Clopidogrel metabolite concentration (μg/mL) and platelet aggregation (AU·min) in patients with genotype *ABCB1 3435C* > *T* before and after PCI

Parameter	Genotype *ABCB1 3435C* > *T*		
	CC	CT	TT
Before PCI	*N* = 40 (25.8%)	*n* = 72 (46.5%)	*n* = 43 (27.7%)
C (μg/mL)	1.13 ± 0.75 (0.08–5.14)	1.32 ± 1.07 (0.06–6.66) NS	1.05 ± 0.65 (0.08–3.12) NS
AUC_agr_ (AU·min)	287.00 ± 152.57 (82–635)	319.86 ± 215.18 (42–1275) NS	299.56 ± 183.68 (61–946) NS
After PCI	*n* = 24 (24%)	*N* = 46 (46%)	*N* = 30 (30%)
C (μg/mL)	1.10 ± 0.52 (0.20–1.92)	1.18 ± 0.67 (0.11–3.24) NS	1.12 ± 0.58 (3.12–2.44) NS
AUC_agr_ (AU·min)	300.64 ± 178.05 (99–899)	313.24 ± 200.92 (55–1137) NS	279.93 ± 220.14 (51–1108) NS

*n* number of patients, *NS* not statistically significant when compared to CC genotype, *p* > 0.05 (Mann–Whitney *U* test)

AUC_agr_—platelet aggregation (AU·min); C—clopidogrel metabolite concentration (μg/mL)

Mean ± SD and (Min–Max) values are presented

**Table 7:** Clopidogrel metabolite concentration (μg/mL) and platelet aggregation (AU·min) in patients with genotype *ABCB1 2677G* > *T,A* before and after PCI

Parameter	Genotype *ABCB1 2677G* > *T,A*			
	GG	GT	TT	GA + TA
Before PCI	*n* = 55 (35.5%)	*n* = 64 (41.3%)	*n* = 30 (19.3%)	*n* = 6 (3.9%)
C (μg/mL)	1.12 ± 0.72 (0.08–3.74)	1.31 ± 1.10 (0.07–6.66) NS	1.21 ± 0.69 (0.06–3.12) NS	0.57 ± 0.42 (0.09–1.27) *p* = 0.026*
AUCagr (AU·min)	308.00 ± 171.56 (82–855)	338.12 ± 234.45 (42–1275) NS	246.66 ± 106.35 (61–514) NS	235.17 ± 109.63 (118–410) NS*
After PCI	*n* = 37 (37%)	*n* = 38 (38%)	*n* = 23 (23%)	*n* = 2 (2%)
C (μg/mL)	1.11 ± 0.62 (0.11–3.23)	1.13 ± 0.61 (3.12–3.24) NS	1.17 ± 0.56 (0.57–2.44) NS	1.40 ± 0.04 (1.37–1.43) *p* = NS*
AUCagr (AU·min)	328.49 ± 176.41 (64–899)	335.21 ± 246.70 (55–1137) NS	235.30 ± 127.15 (51–531) NS	277.00 ± 46.67 (244–310) NS*

*n* number of patients, *NS* not statistically significant when compared to GG genotype, *p* > 0.05 (Mann–Whitney *U* test)

AUC_agr_—platelet aggregation (AU·min); C—clopidogrel metabolite concentration (μg/mL)

Mean ± SD and (Min–Max) values are presented

*For carriers of 2677A allele (GA and TA genotypes) compared to non-carriers of A allele (GG + GT + TT genotypes)

**Table 8:** Clopidogrel metabolite concentration (μg/mL) and platelet aggregation (AU·min) in patients with genotype *ABCB1 1236C* > *T* before and after PCI

Parameter	Genotype *ABCB1 1236C* > *T*		
	CC	CT	TT
Before PCI	*n* = 56 (36.1%)	*n* = 67 (43.2%)	*n* = 32 (20.7%)
C (μg/mL)	1.09 ± 0.72 (0.07–5.14)	1.30 ± 1.08 (0.07–6.66) NS	1.17 ± 0.69 (0.06–3.12) NS
AUC_agr_ (AU·min)	288.40 ± 165.42 (82–855)	343.18 ± 229.22 (42–1275) NS	257.75 ± 125.11 (61–635) NS
After PCI	*n* = 36 (36%)	*n* = 41 (41%)	*n* = 23 (23%)
C (μg/mL)	1.17 ± 0.62 (0.11–3.23)	1.07 ± 0.59 (0.05–3.24) NS	1.20 ± 0.57 (0.57–2.44) NS
AUC_agr_ (AU·min)	330.81 ± 174.82 (64–899)	329.00 ± 241.45 (55–1137) NS	237.40 ± 125.07 (51–531) NS

*n* number of patients, *NS* not statistically significant when compared to CC genotype, *p* > 0.05 (Mann–Whitney *U* test)

AUC_agr_—platelet aggregation (AU·min); C—clopidogrel metabolite concentration (μg/mL)

Mean ± SD and (Min–Max) values are presented

Table 9 shows the association of *PON1192QR* genotype with clopidogrel metabolite concentration and platelet aggregation in patients before and after PCI. There were no significant differences between the inactive metabolite concentration of clopidogrel and platelet aggregation in patients with *the* examined genotype*.*

**Table 9:** Clopidogrel metabolite concentration (μg/mL) and platelet aggregation (AU·min) in patients with genotype *PON1 192QR(A* > *R)* before and after PCI

Parameter	Genotype *PON1 192QR (A* > *G)*		
	QQ	QR	RR
Before PCI	*n* = 83 (53.5%)	*n* = 60 (38.7%)	*n* = 12 (7.8%)
C (μg/mL)	1.26 ± 0.77 (0.04–3.74)	1.13 ± 1.05 (0.06–6.66) NS	1.08 ± 0.81 (0.47–3.28) NS
AUC_agr_ (AU·min)	297.45 ± 172.90 (49–855)	317.33 ± 211.15 (92–1275) NS	305.25 ± 223.02 (42–820) NS
After PCI	*n* = 51 (51%)	*n* = 42 (42%)	*n* = 7 (7%)
C (μg/mL)	1.11 ± 0.65 (0.05–3.24)	1.17 ± 0.50 (0.02–2.44) NS	1.10 ± 0.80 (0.55–2.78) NS
AUC_agr_ (AU·min)	308.12 ± 177.46 (51–899)	299.79 ± 225.83 (55–1137) NS	364.71 ± 185.45 (153–664) NS

*n* number of patients, *NS* not statistically significant when compared to QQ genotype, *p* > 0.05 (Mann–Whitney *U* test)

AUC_agr_—platelet aggregation; C—clopidogrel metabolite concentration

Mean ± SD and (Min–Max) values are presented

### Multivariate analysis

Multivariate analysis (GLM) including age, gender, BMI, and CYP2C19*2 genotype as independent variables and logarithm of platelet aggregation as dependent variable showed that presence of CYP2C19*2 allele is an independent factor associated with significantly higher platelet aggregation in study group both before PCI (*β* =  + 0.18, *p* = 0.019) and after PCI (*β* =  + 0.32, *p* = 0.0022). In this model older age is an independent factor associated with higher platelet aggregation before PCI (*β* =  + 0.23, *p* = 0.0032). Analogical analysis with the logarithm of clopidogrel metabolite concentration as dependent variable showed that the presence of A allele in 2677 position in *ABCB1* gene is an independent factor associated with significantly lower clopidogrel metabolite concentration before PCI (*β* = − 0.18, *p* = 0.022). In that model male gender is an independent factor associated with significantly lower clopidogrel metabolite concentration (*β* = − 0.22, *p* = 0.0049).

## Discussion

Dual antiplatelet therapy with aspirin and P2Y12 inhibitor is recommended for ischemic events after acute coronary syndromes [1–4]. As a potent P2Y12 inhibitor ticagrelol is strongly recommended over clopidogrel to reduce the risk of ischemic recurrences [3]. However, the effective ischemic protection offered by ticagrelol is potentially counterbalanced by an increased risk of bleeding [3, 4]. Recently, increased attention has been focused on preventing bleeding complications in patients on dual antiplatelet therapy, based on evidence showing that risk of death after major bleeding is equal, if not higher than, that following myocardial infarction [4, 5]. Several registries have shown that clopidogrel is frequently preferred by clinicians in patients with acute coronary syndrome [4, 6, 7, 9]. Sub-analysis of the START-ANTIPLATELET registry [4] confirms that clopidogrel, rather than potent P2Y12 inhibitors, is frequently used in real-world practice, especially in acute coronary syndrome patients with high ischemic and bleeding risk. No difference for ischemic and bleeding events was observed in high-bleeding risk patients on clopidogrel versus ticagrelol [4]. Gimbel et al. [31] investigated the safety and efficacy of clopidogrel compared with ticagrelor or prasugrel in older patients with non-ST-elevation acute coronary syndrome (NSTE-ACS). They showed that clopidogrel is an important therapeutic agent for both primary and secondary prevention of coronary heart disease and could be an alternative P2Y12 inhibitor especially for elderly patients with higher bleeding risk.

Despite a large number of studies regarding cardiovascular events in clopidogrel treatment patients, great effort is put to explain the mechanism of treatment resistance and the specificity and sensitivity of the markers helpful in therapy individualization. The active metabolite of clopidogrel is a very labile molecule that degrades rapidly in blood after sample collection, therefore determination of the concentration of the inactive carboxylic acid metabolite of clopidogrel might be useful to evaluate the response to clopidogrel therapy. Serebruany et al. [26] showed that the plasma level of inactive carboxylic acid metabolite of clopidogrel is a useful marker for monitoring compliance to clopidogrel.

Antiplatelet response to clopidogrel and its influence upon the risk of cardiovascular adverse events among patients with stable coronary artery disease undergoing percutaneous coronary intervention (PCI) has been investigated in the same groups of patients from the Department of Cardiology by Olędzki et al. [32]. It has been shown that platelet response to clopidogrel has no impact upon post-procedural adverse events at mid-term follow-up in patients with stable CAD undergoing PCI. This finding suggests that routine platelet reactivity testing is not beneficial in this group of patients.

The aim of the study was to investigate whether the so far studied polymorphisms of genes involved in the metabolism of clopidogrel and its absorption may affect the concentration of the inactive carboxyl metabolite which could be a potential marker for monitoring compliance to clopidogrel. The active thiol metabolite of clopidogrel is unstable in plasma. Its maximum concentration varies between 7.1–13.3 ng/mL. Due to its short half-life, the determination of the concentration of this compound requires specific and sensitive analytical methods. Evaluation of the pharmacokinetics of clopidogrel and its metabolites requires the collection of at least several blood samples. In the clinical setting, it is very difficult. In contrast, the inactive metabolite of clopidogrel occurs in concentrations greater than 1000-fold higher and is present significantly longer in plasma.

The paper presents a study of the influence of selected polymorphisms CYP2C19, CYP 3A4/5, ABCB1 and PON1 on the concentration of inactive metabolite clopidogrel and its pharmacodynamic effect evaluated by platelet aggregation in patients before and after PCI treated clopidogrel with ASA. For this purpose, a simple and reproducible HPLC method with UV detection for the determination of clopidogrel metabolite in human plasma was developed and validated. For HPLC analysis with UV detection a small amount of plasma was used, a reduced amount of acetonitrile relative to the phosphate buffer in the mobile phase. Under the chromatographic conditions described, the drug and I.S. were well resolved in plasma samples and eluted at 1.9 min and 8.9 min, respectively. In the developed method, the retention time of clopidogrel metabolite and I.S. was comparable with the retention time of the inactive clopidogrel metabolite obtained in the method developed by Rouini et al. [33] and Mitakos et al. [34]. However, in the methods developed by Souri et al. [35] and Singh et al. [36] the elution times were significantly longer. The developed method was used to determine concentrations of inactive clopidogrel metabolite measured at steady state in 155 patients qualified for PCI. The time of sampling was determined based on the pharmacokinetic studies conducted so far. It was the time of the peak concentration of both clopidogrel and its active metabolite [28, 36, 37]. In the clinical setting, it was not possible to take more blood samples from the examined patients. The concentration of the inactive metabolite of clopidogrel determined 2 h after administration drug was individually variable and was 0.04 μg/mL to 5.73 μg/mL on average 1.20 μg/mL. Large differences in the carboxylic acid concentrations of clopidogrel demonstrated in our studies were also observed and reported in other authors' reports. The results obtained were comparable to the values of maximum concentrations of inactive metabolite obtained in the Caucasian population [33, 37]. In conclusion, our HPLC method is a simple, specific, reproducible with low-cost determination of non-active metabolite of clopidogrel in plasma of clopidogrel treatment patients. The non-active metabolite of clopidogrel measured in a steady state in a single blood sample is a good marker for drug usage.

One of the most frequently performed analyzes is the influence of CYP2C19 genotypes on the pharmacodynamic effect of clopidogrel**.** It has been shown that CYP2C19*2 variant was associated with poor clopidogrel response and increase ischemic events. However, it has also been confirmed that CYP2C19*17 polymorphism may lead to a higher concentration of clopidogrel active metabolite, an enhanced antiplatelet activity, and an increased risk of bleeding events during clopidogrel therapy [10, 15, 17, 18]. In our study, 40 patients were carriers of the CYP2C19*2 allele. None of the subjects had a homozygous genotype *2/*2 and there was no CYP2C19*3 allele carrier. In patients with CYP2C19*2 allele significantly reduced anti-aggregation effect after drug administration was observed. The incidence of at least one CYP2C19*2 and CYP2C19*3 allele responsible for disturbed metabolism of clopidogrel in the Caucasian population is estimated at 5–30% for CYP2C19*2 and less than 1% for CYP2C19*3 [10, 38, 39]. In studied group 115 subjects were fast metabolizers (EM) with *1/*1, *1/*17, *17/*17 genotypes and 40 subjects were intermediate metabolizers (IM) with *1/*2 and *2/*17 genotypes. In EM group platelet aggregation was reduced by 58% in comparison to values obtained in IM group (*p* = 0.0006). The concentrations of inactive metabolite were not significantly increased in IM compared to EM group which could suggest that less of the drug was metabolized to the active metabolite. This suggestion may be confirmed by a decreased antiplatelet effect of clopidogrel in this group. Li et al. [40] showed that in subjects with ultrafast metabolism which is associated with *17/*17 genotype and in subjects with at least one *17 allele recurrent cardiovascular events were less frequently observed, but the bleeding was more frequent during the use of clopidogrel treatment. This effect of the study has been confirmed by other authors [15, 39]. In our study 44 subjects were *1/*17 allele carriers, 18 subjects had *17/*17 genotype and 15 patients *2/*17 genotype. In studied group, no significant differences regarding to the effect of CYP2C19*17 alleles on the concentration of inactive metabolite and pharmacodynamic effect of clopidogrel in EM (1*/*17 and 17*/*17), whereas in carriers of *2/*17 and *1/*2 (IM) genotypes the platelet aggregation was significantly higher. Sibbing et al. [15] suggest that in the haplotype *2/*17, where one allele encodes the impaired enzyme activity and the other allele is responsible for ultrafast metabolism, decreased CYP2C19 activity due to the presence of the CYP2C19 *2 variant is dominant.

It has been shown that clopidogrel absorption is limited by the intestinal efflux transporter P-glycoprotein encoded by *ABCB1* gene. Several trials have shown that *ABCB1* 3435T was associated with lower levels of plasma clopidogrel and its active metabolite [10, 23, 41, 42]. However, there are also inconsistent reports on the association of ABCB1 polymorphism and clopidogrel response. We analyzed 3435C > T, 2677G > T,A and 1236C > T *ABCB1* gene polymorphisms. Similarly to other authors, we did not find any association between CYP3A4*22, CYP3A5*3, *ABCB1* 3435C > T and 1236C > T and *PON1* 192QR and concentration of inactive clopidogrel metabolite or platelet aggregation in patients before and after PCI [22, 38, 42–45]. We conclude that plasma inactive metabolite concentration and platelet reactivity depend mainly on the CYP2C19 polymorphism.

The determination of the concentration of the inactive metabolite simultaneously with platelet aggregation could be used in clinical practice in patients chronically treated with clopidogrel especially in acute coronary syndrome patients with high ischemic and bleeding risk or in patients with no treatment effect.
